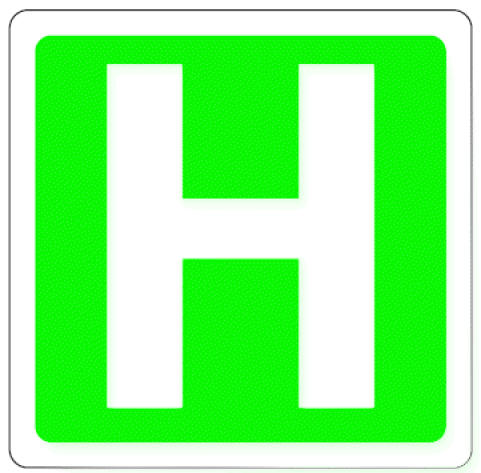# The Beat

**Published:** 2005-02

**Authors:** Erin E. Dooley

## NASA for Nature

In November 2004 the World Conservation Union (IUCN) and NASA announced the formation of a partnership to use NASA’s data, images, and remote sensing capabilities to help the IUCN in its efforts to conserve biodiversity around the world. NASA data will be used in support of several IUCN programs, including the Protected Areas Learning Network and the World Database on Protected Areas. Many protected areas are remote and in regions with few resources, and so are poorly mapped. But together with NASA, the IUCN will be able to generate satellite imagery and accurate maps to help create a protected area archive.

## Global Warming On the Streets

The British government launched an advertising campaign in October 2004 to educate the public about the potential dangers of climate change. The £6 million campaign kicked off with the publication of a report detailing the effects that could result from higher temperatures. The ads themselves suggest ways the public can help curb global warming—for example, by turning down their thermostats, choosing cars that run on renewable energy, and properly insulating their homes. The advertisements will appear in the press, on billboards, and possibly on television. The British Energy Saving Trust estimates that Brits currently waste £5 billion worth of energy each year.

## Millennium Development Progress Report

The United Nations has issued a progress report on the Millenium Development Goals, which were set in September 2000 in an attempt to halve world poverty by 2015. Most countries appear on track to halve the number of people without access to safe water (although 800 million would still be at risk), but progress toward meeting sanitation goals is slow or nonexistent in several regions. By 2015, about 2.4 billion people could still face disease and death due to poor sanitation, which contributes to the spread of cholera and diarrheal disease, killing a child every 21 seconds. Western and southern Asia, sub-Saharan Africa, and Oceania are among the regions showing the least progress; conditions are deteriorating in some developed countries such as the former Soviet republics.

## Machu Picchu Makeover

The World Bank announced a $5 million loan in September 2004 to go toward protecting the area around Machu Picchu, one of Peru’s main tourist and cultural areas and a World Heritage Site. The Peruvian government is contributing an additional $3.2 million for the project, which will work to develop sustainable tourism initiatives, reduce pollution at the site, and improve environmental management in the Vilcanota Valley, where the site is located. The project will also improve the region’s solid waste management system and allow for detailed environmental impact assessments to analyze the effect of urbanization of the area. The new water and sanitation developments may employ technologies such as solar aquatic systems and wastewater gardens. The local community has been consulted extensively in developing the project.

## World Progress Up in Smoke?

*Up in Smoke*, released in October 2004 by the Working Group on Climate Change and Development, asserts that global warming could make it impossible to achieve the Millenium Development Goals set in 2000 by the United Nations, and could even reverse progress that has been made toward meeting the goals. The report states that the impacts of climate change will fall disproportionately on the poor in both developed and developing countries, and provides many scenarios to illustrate. For example, in developing regions where food security is already tenuous, increased flooding related to global warming could wipe out plant resources, stored food, and seed reserves. The report states that industrialized countries need to surpass Kyoto Protocol goals and cut greenhouse gas emissions by 60–80% below 1990 levels in order to stop climate change from “running out of control.”

## Green Guide for Health Care

The first-ever sustainable design guidebook for hospitals was released on 22 November 2004 by the Austin, Texas–based Center for Maximum Potential Building Systems. The product of a two-year multistakeholder development process, the guide is based on the U.S. Green Building Council’s Leadership in Energy and Environmental Design rating system but has been modified to meet the specific needs of the hospital industry from start to finish. The program addresses topics including round-the-clock energy performance, chemical use, infection control requirements, and the elimination of toxic chemicals from building materials. Several major hospital systems, including Kaiser-Permanente, have agreed to pilot-test the guide over the next year.

## Figures and Tables

**Figure:**
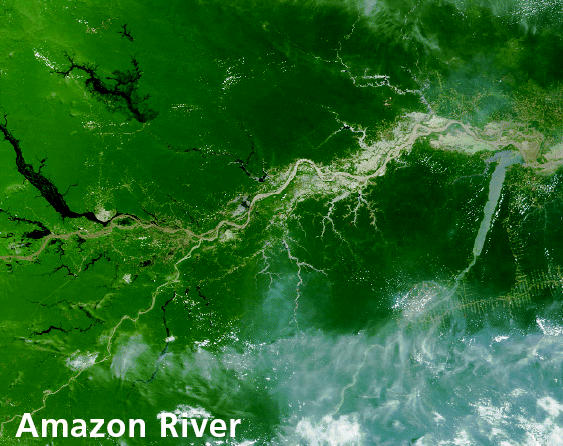


**Figure:**
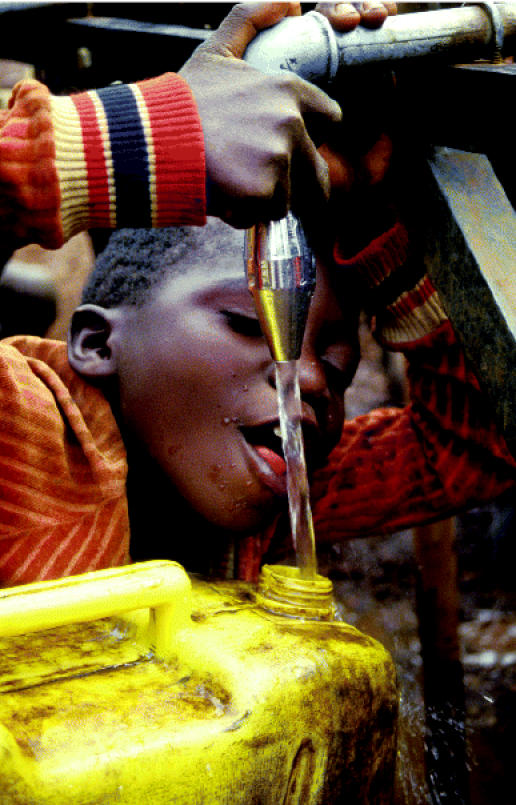


**Figure:**
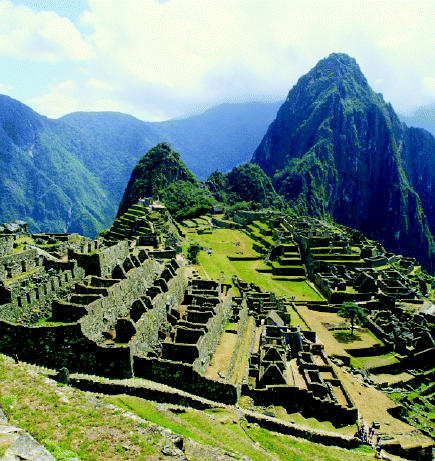


**Figure:**